# Early life risk factors for childhood obesity—Does physical activity modify the associations? The MoBa cohort study

**DOI:** 10.1111/sms.13504

**Published:** 2019-07-03

**Authors:** Guro Pauck Bernhardsen, Trine Stensrud, Wenche Nystad, Knut Eirik Dalene, Elin Kolle, Ulf Ekelund

**Affiliations:** ^1^ Department of Sport Medicine Norwegian School of Sports Sciences Oslo Norway; ^2^ Department of Non‐communicable Diseases Norwegian Institute of Public Health Oslo Norway

**Keywords:** birth weight, childhood BMI, childhood body composition, infant weight gain, maternal pre‐pregnancy BMI, physical activity

## Abstract

**Objectives:**

High maternal pre‐pregnancy body mass index (BMI), high birth weight, and rapid infant weight gain are associated with increased risk of childhood obesity. We examined whether moderate‐to‐vigorous physical activity (MVPA) or vigorous physical activity (VPA) in 9‐ to 12‐year‐olds modified the associations between these early life risk factors and subsequent body composition and BMI.

**Methods:**

We used data from a sub‐cohort of the Norwegian Mother and Child Cohort Study (MoBa), including 445 children with available data on accelerometer assessed physical activity (PA). All participants had data on BMI, 186 of them provided data on body composition (dual energy X‐ray absorptiometry (DXA)). We used multiple regression analyses to examine the modifying effect of PA by including interaction terms.

**Results:**

Maternal pre‐pregnancy BMI and infant weight gain were more strongly related to childhood body composition in boys than in girls. Higher VPA attenuated the association between maternal pre‐pregnancy BMI and BMI in boys (low VPA: *B* = 0.32, 95% CI = 0.22, 0.41; high VPA *B* = 0.22, 95% CI = 0.12, 0.31). Birth weight was unrelated to childhood body composition, and there was no effect modification by PA. PA attenuated the associations between infant weight gain and childhood fat mass (low MVPA:* B* = 2.32, 95% CI = 0.48, 4.17; high MVPA: *B* = 1.00, 95% CI = 0.10, 1.90) and percent fat (low MVPA: *B* = 3.35, 95% CI = 0.56, 6.14; high MVPA: *B* = 1.41, 95% CI = −0.06, 2.87) in boys, but not girls.

**Conclusion:**

Findings from this study suggest that MVPA and VPA may attenuate the increased risk of an unfavorable body composition and BMI due to high maternal pre‐pregnancy BMI and rapid infant weight gain in boys, but not in girls.

## 1 INTRODUCTION

Childhood obesity is related to several short‐term health consequences^1^ and is a strong predictor of adult obesity with the accompanying risks of cardiovascular diseases and mortality.^2^, ^3^ The development of obesity may start before birth, with intrauterine and early life exposures having long‐term effects on biology, leading to an increased fat mass and risk of obesity later in life.^4^, ^5^ Previous research has established high maternal pre‐pregnancy body mass index (BMI), high birth weight, and rapid infant weight gain as risk factors for childhood obesity.^1^, ^6^, ^7^, ^8^ Maternal pre‐pregnancy obesity is associated with three times increased odds for childhood overweight or obesity.^9^ Similarly, high birth weight (>4.0 kg) is associated with a twofold increased odds of obesity compared to normal birth weight (2.5‐4.0 kg).^10^ Both high and low birth weight have been linked to subsequent development of obesity and non‐communicable diseases.^11^ Furthermore, low birth weight, reflecting under‐nourishment and fetal growth restriction, is often accompanied by rapid postnatal weight gain, which is an independent additional risk factor for later obesity,^6^ with a nearly twofold increase in the odds of childhood obesity in those children increasing their weight by at least one standard deviation (*z*‐score) between birth and 1 year.^6^

Body mass index is frequently used to define overweight and obesity. However, using BMI as a proxy for adiposity has obvious limitations due to its inability to differentiate between fat mass and fat‐free mass. In studies where detailed measurements of body composition are available, higher maternal pre‐pregnancy BMI, higher birth weight, and rapid infant weight gain are associated with subsequent higher fat mass in childhood.^12^, ^13^, ^14^, ^15^

Furthermore, previous studies suggest that physical activity (PA), especially high intensity PA, is associated with lower fat mass in children.^16^, ^17^ Hence, PA may be an important strategy to prevent an unfavorable body composition, particularly in those children prone to higher adiposity as a result of high maternal pre‐pregnancy BMI, high birth weight, or rapid infant weight gain. Moreover, it could be hypothesized that higher PA may attenuate the positive associations between maternal pre‐pregnancy BMI, birth weight and infant weight gain, and subsequent adiposity.

Currently, the evidence is inconclusive. One study suggested that PA does not modify the associations between birth weight, fat mass index (fat mass/m^2^), and waist circumference in 9‐ and 15‐year‐olds.^18^ In contrast, others have suggested an effect modification on the association between birth weight and higher risk of obesity by self‐reported moderate‐to‐vigorous physical activity (MVPA) in girls, but not boys.^19^ Kolle et al^20^ examined the associations between weight gain in infancy (0‐2 years) and childhood (2‐4 years) with subsequent fat mass in 30‐year‐olds and observed that the association between weight gain in childhood and fat mass index in adulthood was attenuated by objectively measured MVPA. No effect modification of MVPA was observed on the association between infant weight gain and subsequent fat mass index. Thus, additional research is needed to examine whether PA mitigates the associations between early life exposures and long‐term risk of obesity.

Examining the modifying effect of PA on early life obesity risk factors is important to obtain a more comprehensive understanding of the underlying biological processes for obesity development and for potentially efficacious prevention strategies. The aim of this study was to examine if MVPA or vigorous PA (VPA) in childhood modifies the associations between maternal pre‐pregnancy BMI, birth weight, and infant weight gain with precisely measured body composition and BMI in 9‐ to 12‐year‐old Norwegian boys and girls.

## 2 METHODS

### 2.1 Study design and participants

We used data from a sub‐cohort of the Norwegian Mother and Child Cohort Study (MoBa). MoBa is an ongoing prospective population‐based cohort study managed by the Norwegian Institute of Public Health (NIPH).^21^ All women attending a routine ultrasound examination at 17‐20 weeks gestation at Norwegian maternity units that deliver more than 100 births annually (50 units out of 52 participated between 1999 and 2008) were invited to participate. More than 100 000 children have been followed‐up from birth. In this study, we used data from a sub‐cohort of 1603 participants born between 2002 and 2004, living within a 1‐hour radius from one of four test centers. These participants were invited to undergo additional testing, including anthropometric, body composition, and PA measurements. The tests were conducted between 2013 and 2015, in Oslo, Bergen, Stavanger and Fredrikstad. A total of 470 children agreed to participate (participation rate 29.3%), of which 445 participants provided complete PA data (flow‐chart of participants in Figure S1). A dual X‐ray absorptiometry (DXA) scan was only available at the test center located in Oslo; thus, body composition measurements were only performed in 186 participants. Therefore, the number of participants included in different analyses differs depending on the outcome measure.

The establishment of MoBa and initial data collection was based on a license from the Norwegian Data protection agency and the approval from The Regional Committee for Medical Research Ethics. The MoBa cohort is currently regulated by the Norwegian Health Registry Act. The present sub‐study was additionally approved by The Regional Committee for Medical Research Ethics (REC South East). A written informed consent was obtained from the children's parents prior to all measurements.

### 2.2 Measurements

#### 2.2.1 Exposures

The mothers reported their pre‐pregnancy weight and height in gestational week 18. We obtained birth weight and gestational age at birth which was estimated using ultrasound, from the Medical Birth Registry of Norway (MBRN). MBRN is a national health registry containing information about all births in Norway. We standardized birth weight as sex‐ and gestational age‐specific *z*‐scores using information from more than 100 000 births in the MoBa cohort. The mothers reported their children's weight at 1‐year via questionnaire. We advised the mothers to use the measured weight recorded on their child's health record. We calculated infant weight gain as change in sex‐specific *z*‐scores from birth to 1 year, using the mean and standard deviation (SD) from the entire MoBa cohort.

#### 2.2.2 Outcomes

The participants and their parents were asked to attend their nearest test center where trained personnel performed body composition and anthropometric measurements. The participants wore light clothing during body weight and height measurements, which were performed using a mechanical scale and a stadiometer, respectively. We calculated BMI by dividing weight by height squared (kg/m^2^).

Trained personnel performed the DXA‐measurements using a Lunar iDXA (GE Healthcare Lunar) (enCORE Pediatric whole‐body analysis Software Version 14.10.022). The participants underwent a whole‐body scan, wore light clothes, and removed all loose metal items prior to scanning. The test personnel calibrated the scanner daily according to the Lunar iDXA enCORE manual.

#### 2.2.3 Covariates

Parental education was self‐reported via questionnaire at gestational week 18. We estimated parental education as the highest completed or ongoing education by either the mother or the father.

We also modeled measured birth weight and self‐reported maternal pre‐pregnancy BMI as covariates when not modeled as the exposure of interest.

For descriptive statistics, we defined childhood underweight, overweight, and obesity using the International Obesity Task Force (IOTF) criteria, whereby “underweight” corresponds to an adult BMI value of ≤18.5 kg/m^2^, “overweight” corresponds to an adult BMI value of ≥25kg/m^2^, and “obesity” corresponds to an adult BMI value of ≥30 kg/m^2^.^22^, ^23^

#### 2.2.4 Effect modifier—physical activity

We measured PA using Actigraph accelerometers (Actigraph GT3X+; LLC), previously validated in free living conditions among children.^24^ The participants wore the monitor on their right hip and were instructed to wear the accelerometer during all waking hours for seven consecutive days (in addition to the day the monitor was attached), except while bathing or doing other water‐based activities. To eliminate reactivity bias, we set the monitors to start recording at 06:00 the day after the participants received the monitors.^25^ We used ActiLife (version 6.13.3) to process the data and used an epoch length of 10 seconds. We excluded overnight activity (00:00‐05:59) from our analyses and defined non‐wear time as 20 minutes or more of consecutive zero counts. A day was considered valid if the participant wore the monitor for at least 480 minutes. Ninety‐eight percent of the participants provided three or more valid days of activity recordings, and there was no difference in MVPA and VPA between these children and those who provided less than three valid day. Ninety‐four percent of the participants provided at least one valid weekday and one weekend, with no difference in MVPA and VPA compared to those children that did not. Hence, all participants providing at least one valid day were included in the analyses. MVPA (min/d) was defined as ≥2296 counts per minute (cpm), whereas VPA (min/d) was defined as ≥4012 cpm,^26^ as recommended to estimate PA intensities in children.^27^

### 2.3 Statistical analyses

Descriptive statistics of participants are presented as mean and SD for continuous variables and number of participants and proportions (%) for categorical variables (parental education and BMI classification). We tested for differences between boys and girls using independent samples *t* tests for continuous variables, Mann‐Whitney two‐sample tests for parental education and chi‐squared tests for categorical data (underweight, normal weight, overweight, and obese). We used multiple regression to examine the associations between early life measurements (maternal pre‐pregnancy BMI, birth weight *z*‐score, and infant weight gain), childhood body composition, and BMI. We examined for linearity between independent and dependent variables and each model for normal distribution of residuals and homoscedasticity. We included a sex interaction term as previous studies have observed sex‐dependent associations.^19^, ^28^ The formal interaction tests showed that certain associations may differ between boys and girls (*P* < 0.05), thus we stratified all analyses by sex. We adjusted each model for parental education. When PA and BMI were modeled as the outcome we additionally adjusted for current age, whereas models including body composition (ie fat mass, percent fat, and fat‐free mass) as the outcome measure were additionally adjusted for current height. Furthermore, other possible covariates were included depending on the exposure. For example, when maternal pre‐pregnancy BMI and infant weight gain were modeled as the exposures, we additionally adjusted the analyses for birth weight. Similarly, the models including birth weight as the main exposure were adjusted for maternal pre‐pregnancy BMI. To test if MVPA or VPA modified the associations, we included the interaction terms; early life risk factor × MVPA or early life risk factor × VPA into separate models. In the case of a significant interaction, we graphically displayed the predicted values of the outcome variable (calculated from the adjusted regression models with the interaction terms), at given values of the exposure of interest and the 25th, 50th, and 75th percentile of MVPA and VPA. In addition, we stratified the participants using a median split in MVPA and VPA and examined the magnitude of the associations in the models without the interaction term.

The number of participants with missing values for one or more variables was 50 (20.7%) and 37 (18.2%) for boys and girls, respectively. We replaced missing values on exposures and covariates using fully conditional specification (FCS) multiple imputation (predictive mean matching and ordered logistic regression). We imputed a total of 20 datasets separately for boys and girls, based on all variables in the full models in addition to auxiliary variables. More information about the imputation method, imputation model, number of missing values for each variable, and complete case analyses are provided in File S1.

The statistical significance level was 5% for all analyses. We used Stata/SE version 14.2 to conduct the analyses.

## 3 RESULTS

Table 1 summarizes the characteristics of the total study sample and our sub‐group with available DXA measurements. Overall, boys had a significantly higher birth weight and weight at 1 year than girls, whereas girls had higher fat mass and percent fat mass in childhood. On average, participants wore the accelerometer for 6.5 (SD 1.34) days, and for 786 (SD 55) min/d. Boys spent significantly more time in MVPA and VPA than the girls.

**Table 1:** Characteristics of the study participants at birth and follow‐up (9 to 12 y old). All values are mean (SD) unless otherwise specified

	All participants		Participants with DXA	
	Boys (n = 242)	Girls (n = 203)	Boys (n = 98)	Girls (n = 88)
Age (y)	10.9 (0.66)	10.9 (0.66)	10.9 (0.63)	11.0 (0.63)
Birth weight (kg)	3.70 (0.62)*	3.58 (0.54)*	3.76 (0.62)*	3.54 (0.58)*
Gestational age (wk)	39.5 (1.96)	39.5 (1.52)	39.8 (1.84)	39.5 (1.84)
Weight 1 y (kg)	10.30 (1.01)*	9.59 (0.99)*	10.43 (1.07)*	9.56 (1.04)*
Maternal pre‐pregnancy BMI (kg/m^2^)	23.9 (4.3)	23.8 (4.1)	23.7 (4.1)	24.0 (3.9)
Parental education^a^, n (%)				
<High school	6 (2.5%)	4 (2.0%)	2 (2.1%)	3 (3.5%)
High school	50 (20.9%)	48 (24.0%)	18 (18.7%)	13 (15.1%)
College/university 1‐4 y	95 (39.7%)	80 (40.0%)	40 (41.7%)	36 (41.9%)
College/university > 4 y	88 (36.8%)	68 (34.0%)	36 (37.5%)	34 (39.5%)
Weight (kg)	39.2 (7.3)	39.1 (7.7)	39.2 (6.9)	40.0 (8.4)
Height (m)	1.47 (0.07)	1.48 (0.08)	1.48 (0.06)	1.49 (0.08)
Fat mass (kg)	NA	NA	10.0 (4.19)*	11.5 (4.6)*
Fat‐free mass (kg)	NA	NA	29.4 (3.56)	28.6 (4.84)
Percent fat mass (%)	NA	NA	24.7 (6.15)*	28.1 (5.76)*
BMI (kg/m^2^)	17.9 (2.47)	17.7 (2.30)	17.8 (2.46)	17.9 (2.33)
Underweight (yes), n (%)	18 (7.4%)	18 (8.9%)	9 (9.2%)	4 (4.5%)
Normal weight (yes), n (%)	189 (78.1%)	161 (79.3%)	76 (77.5%)	72 (81.8%)
Overweight/obesity (yes), n (%)	35 (14.5%)	24 (11.8%)	13 (13.3%)	12 (13.6%)
Obesity (yes), n (%)	5 (2.1%)	1 (0.5%)	3 (3.1%)	1 (1.14%)
SED (min/d)	496 (59.0)	506 (57.3)	501 (61.6)	508 (55.6)
MVPA (min/d)	74 (26.5)*	58 (19.0)*	75 (26.9)*	59 (17.4)*
VPA (min/d)	29 (14.6)*	21 (10.3)*	30 (15.7)*	21 (8.7)*

Abbreviations: BMI, Body mass index (weight/height^2^); MVPA, Moderate‐to‐vigorous physical activity; NA, Not available; SED, Sedentary time; VPA, Vigorous physical activity.

a The education level of the parent with the highest completed or ongoing education (mother or father).

* *P* < 0.05 for difference between boys and girls.

Tables 2 and 3 show the results from the multiple regression analyses examining the associations between exposures and outcomes and the effect modification by physical activity for boys and girls, respectively.

**Table 2:** Unstandardized regression coefficients, with 95% CI, for the associations between MVPA, VPA and early life exposures with body composition measures and BMI in 9 to 12‐year‐old boys, and interaction between early life exposures and MVPA/VPA (in separate models)

	Fat mass (kg)	Fat‐free mass (kg)	Percent fat (%)	BMI (kg/m^2^)
	n = 98	n = 98	n = 98	n = 242
MVPA (min/d)^a^	**−0.03 (−0.06, −0.00)**	−0.01 (−0.03, 0.02)	−0.05 (−0.09, 0.00)	**−0.01 (−0.03, −0.00)**
VPA (min/d)^a^	**−0.05 (−0.11, −0.00)**	−0.01 (−0.05, 0.03)	**−0.09 (−0.16, −0.01)**	**−0.03 (−0.06, −0.01)**
Maternal pre‐pregnancy BMI (kg/m^2^)^b^	**0.45 (0.28, 0.63)**	**0.14 (0.02, 0.25)**	**0.64 (0.36, 0.93)**	**0.28 (0.22, 0.35)**
× MVPA (interaction term)	−0.007 (−0.02, 0.00)	−0.005 (−0.01, 0.00)	−0.008 (−0.02, 0.01)	−0.001 (−0.00, 0.00)
× VPA (interaction term)	−0.010 (−0.02, 0.00)	−0.006 (−0.02, 0.00)	−0.014 (−0.04, 0.01)	**−0.005 (−0.01, −0.00)**
Birth weight (*z*‐score)^c^, ^d^	0.24 (−0.45, 0.93)	0.07 (−0.37, 0.51)	0.34 (−0.76, 1.44)	0.13 (−0.14, 0.40)
× MVPA (interaction term)	0.010 (−0.01, 0.03)	0.000 (−0.01, 0.01)	0.031 (−0.00, 0.06)	0.000 (−0.01, 0.01)
× VPA (interaction term)	0.012 (−0.02, 0.05)	0.004 (−0.02, 0.03)	0.039 (−0.01, 0.09)	0.001 (−0.01, 0.01)
Infant weight gain (*z*‐score)^b^, ^e^	**1.45 (0.59, 2.31)**	**0.77 (0.26, 1.28)**	**2.09 (0.74, 3.43)**	**0.75 (0.38, 1.11)**
× MVPA (interaction term)	**−0.026 (−0.05, −0.01)**	−0.003 (−0.01, 0.01)	**−0.035 (−0.06, −0.00)**	0.000 (−0.01, 0.01)
× VPA (interaction term)	**−0.068 (−0.10, −0.02)**	−0.005 (−0.03, 0.02)	**−0.085 (−0.14, −0.03)**	−0.002 (−0.02, 0.02)

Note

Significant results are highlighted in bold letters.

Abbreviations: BMI, Body mass index; MVPA: Moderate‐to‐vigorous physical activity; VPA, Vigorous physical activity.

a Adjusted for highest parental education and current age.

b Adjusted for highest parental education, birth weight and current height (for body composition outcomes only) and current age (for BMI outcome only).

c Adjusted for highest parental education, maternal pre‐pregnancy BMI and current height (for body composition outcomes only) and current age (for BMI outcome only).

d Sex and gestational age‐specific *z*‐scores.

e Change in sex‐specific *z*‐scores from birth to 1 y.

**Table 3:** Unstandardized regression coefficients, with 95% CI, for the associations between MVPA, VPA and early life exposures with body composition measures and BMI in 9 to 12‐year‐old girls, and interaction between early life exposures and MVPA/VPA (in separate models)

	Fat mass (kg)	Fat‐free mass(kg)	Percent fat (%)	BMI (kg/m^2^)
	n = 88	n = 88	n = 88	n = 203
MVPA (min/d)^a^	−0.03 (−0.08, 0.03)	−0.01 (−0.06, 0.05)	−0.02 (−0.09, 0.05)	−0.00 (−0.02, 0.01)
VPA (min/d) a	−0.04 (−0.15, 0.07)	−0.00 (−0.11, 0.11)	−0.04 (−0.18, 0.10)	−0.01 (−0.04, 0.02)
Maternal pre‐pregnancy BMI (kg/m^2^)^b^	0.20 (−0.04, 0.45)	0.07 (−0.05, 0.20)	0.25 (−0.10, 0.61)	**0.10 (0.02, 0.18)**
× MVPA (interaction term)	−0.003 (−0.02, 0.01)	−0.003 (−0.01, 0.01)	0.001 (−0.02, 0.02)	−0.000 (−0.00, 0.00)
× VPA (interaction term)	−0.006 (−0.03, 0.02)	−0.003 (−0.02, 0.01)	−0.000 (−0.04, 0.04)	−0.003 (−0.01, 0.00)
Birth weight (*z*‐score)^c^, ^d^	0.25 (−0.58, 1.09)	0.39 (−0.05, 0.84)	0.21 (−1.00, 1.41)	**0.34 (0.05, 0.64)**
× MVPA (interaction term)	0.017 (−0.04, 0.07)	0.015 (−0.01, 0.04)	0.036 (−0.04, 0.11)	0.000 (−0.01, 0.02)
× VPA (interaction term)	−0.001 (−0.11, 0.11)	0.033 (−0.03, 0.09)	0.024 (−0.14, 0.19)	−0.005 (−0.04, 0.03)
Infant weight gain (*z*‐score)^b^, ^e^	0.04 (−1.24, 1.33)	−0.16 (−0.75, 0.44)	0.28 (−1.38, 1.94)	0.37 (−0.06, 0.81)
× MVPA (interaction term)	−0.021 (−0.08, 0.04)	0.002 (−0.02, 0.03)	−0.041 (−0.12, 0.03)	0.003 (−0.02, 0.02)
× VPA (interaction term)	−0.025 (−0.17, 0.12)	0.005 (−0.06, 0.07)	−0.072 (−0.25, 0.10)	0.013 (−0.03, 0.05)

Note

Significant results are highlighted in bold letters.

Abbreviations: BMI, Body mass index; MVPA: Moderate‐to‐vigorous physical activity; VPA, Vigorous physical activity.

a Adjusted for highest parental education and current age.

b Adjusted for highest parental education, birth weight and current height (for body composition outcomes only) and current age (for BMI outcome only).

c Adjusted for highest parental education, maternal pre‐pregnancy BMI and current height (for body composition outcomes only) and current age (for BMI outcome only).

d Sex and gestational age‐specific *z*‐scores.

e Change in sex‐specific *z*‐scores from birth to 1 y.

The results from the complete case analyses are provided in File S1, Table S3. The effect estimates are similar, but some confidence intervals are wider, in the complete case analyses.

### 3.1 Maternal pre‐pregnancy BMI

A higher maternal pre‐pregnancy BMI was associated with higher fat mass, fat‐free mass, percent fat, and BMI in boys. For example, a 1‐unit increase in maternal BMI was associated with a 0.45 kg (95% CI = 0.28, 0.63) higher childhood fat mass and a 0.64% (95% CI = 0.36, 0.93) higher percent fat (Table 2). There were no significant associations between maternal pre‐pregnancy BMI and the body composition measures in girls (Table 3). The association with BMI was weaker in girls than in boys. There was no evidence that these associations differed according to time spent in MVPA or VPA in girls. However, the test for interaction showed that the association between maternal pre‐pregnancy BMI and childhood BMI was modified by time spent in VPA in boys, indicating that higher levels of VPA attenuated the association.

**Figure 1:**
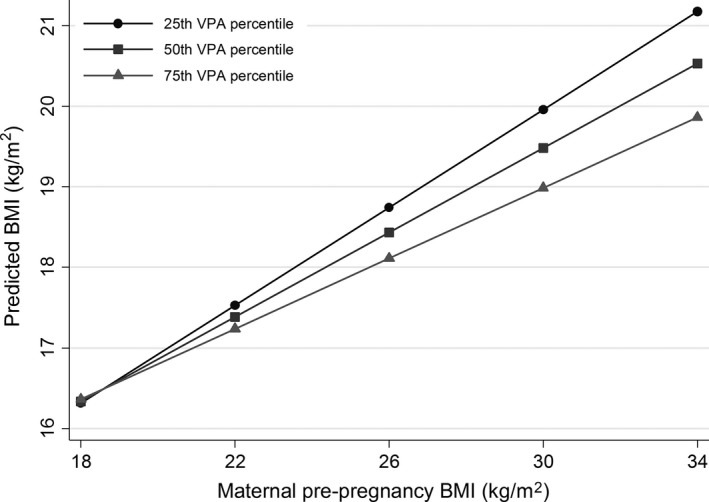
Predicted childhood BMI across values of maternal pre‐pregnancy BMI and the 25th, 50th, and 75th percentile of VPA, in 9‐ to 12‐year‐old boys. Predicted values calculated from multiple regression model with interaction term (maternal pre‐pregnancy BMI × VPA). 25th VPA percentile = 19.7 min/d, 50th VPA percentile = 28.3 min/d, 75th VPA percentile = 37.2 min/d. Adjusted for birth weight, parental education and age. BMI, Body mass index; VPA, Vigorous physical activity

Figure 1 shows the predicted values of the children's BMI at given values of maternal pre‐pregnancy BMI and the 25th, 50th, and 75th percentile of VPA. For example, a maternal pre‐pregnancy BMI of 30 kg/m^2^ (corresponding to mother being obese) gives predicted values of 20.0, 19.5, and 19.0 kg/m^2^ at the 25th, 50th, and 75th percentile of VPA, respectively.

The association between maternal pre‐pregnancy BMI and childhood BMI was stronger (*B* = 0.32, 95% CI = 0.22, 0.41) in the participants below the median VPA (<28 minutes per day), compared with those above (*B* = 0.22, 95% CI = 0.12, 0.31).

### 3.2 Birth weight *z*‐score

The gestational age‐ and sex‐specific birth weight *z*‐scores were not associated with fat mass, fat‐free mass, or percent fat mass in boys or girls (Tables 2 and 3). We observed a positive association between birth weight and BMI in childhood in girls, whereby 1 *z*‐score higher birth weight was associated with 0.34 (95% CI = 0.05, 0.64) higher BMI (Table 3). Including the interaction terms birth weight × MVPA and birth weight × VPA into the models showed no evidence of effect modification by PA (Tables 2 and 3).

### 3.3 Infant weight gain

Change in weight *z*‐scores from birth to 1 year was positively associated with the different components of body composition and BMI in boys (Table 2). For example, a 1‐unit increase in *z*‐score from birth to 1‐year was associated with 1.45 kg (95% CI = 0.59, 2.31) higher childhood fat mass in boys. We did not observe any associations between infant weight gain and adiposity measures in girls (Table 3). MVPA and VPA attenuated the associations between infant weight gain, fat mass, and percent fat in boys.

**Figure 2:**
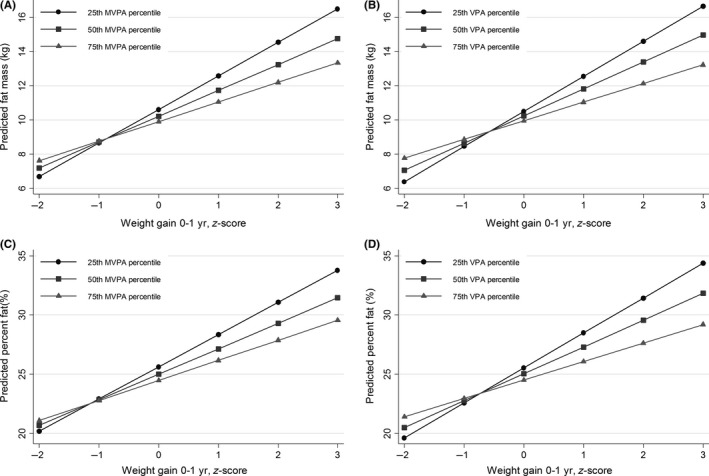
Predicted childhood fat mass (A and B) and percent fat (C and D) across values of infant weight gain and the 25th, 50th, and 75th percentile of MVPA (A and C) and VPA (B and D), in 9‐ to 12‐year‐old boys. Predicted values calculated from multiple regression models with interaction terms (infant weight gain × MVPA/VPA). 25th MVPA percentile = 55.3 min/d, 50th MVPA percentile = 73.8 min/d, 75th MVPA percentile = 88.8 min/d. 25th VPA percentile = 19.7 min/d, 50th VPA percentile = 28.3 min/d, 75th VPA percentile = 37.2 min/d. Adjusted for birth weight, height, and parental education. MVPA, Moderate‐to‐vigorous physical activity; VPA, Vigorous physical activity

Figure 2 shows the predicted values of fat mass and percent fat across infant weight gain *z*‐scores and the 25th, 50th, and 75th percentile of MVPA and VPA in boys. The predicted fat mass in boys given a 0.67 increase in weight *z*‐score from birth to 1‐year (corresponding to upward centile crossing on standard infant growth charts) were 11.9 kg, 11.3 kg, and 10.7 kg in the 25th, 50th, and 75th percentile of VPA, respectively. Similarly, the predicted percentage body fat for a 0.67 increase in weight *z*‐score was 27.5%, 26.6%, and 25.6%, for the 25th, 50th, and 75th percentile of VPA, respectively.

In median split analyses, the association between infant weight gain and fat mass was stronger in the low MVPA group (*B* = 2.32, 95% CI = 0.50, 4.17) compared with the high (*B* = 1.00, 95% CI = 0.10, 1.91) MVPA group (high MVPA > 73.6 min/d). The association with percent fat mass in those below the median for MVPA was *B* = 3.35(95% CI = 0.55, 6.14) compared with those above the median *B* = 1.41(95% CI = −0.06, 2.87).

## 4 DISCUSSION

Our results indicate that high maternal pre‐pregnancy BMI and rapid infant weight gain are stronger predictors for childhood fat mass and BMI in boys than in girls. Furthermore, it appears that some of these associations are attenuated by PA in boys. The results also indicate that birth weight is not associated with body composition in either sexes, nor modified by MVPA or VPA.

A 1‐unit higher maternal pre‐pregnancy BMI was associated with 0.28 (95% CI = 0.22, 0.35) higher BMI in 9‐ to 12‐year‐old boys and 0.10 (95% CI = 0.02, 0.18) higher BMI in girls. MVPA and VPA did not modify this association in girls. Conversely, in boys the magnitude of the association appears to be contingent on VPA, suggesting that VPA might mitigate the increased risk of high childhood BMI that accompanies a higher maternal pre‐pregnancy BMI. The predicted BMI in childhood given a maternal pre‐pregnancy BMI of 30kg/m^2^ was 0.9 higher in the 25th compared to 75th percentile of VPA. This difference is equivalent to a difference of about 17 minutes of VPA between the 25th and the 75th quartile. The association between maternal pre‐pregnancy BMI and childhood BMI is attenuated in those above the median in VPA, although not fully eliminated. These results suggest that a large amount of VPA is necessary to mitigate the increased risk of higher childhood BMI associated with high maternal pre‐pregnancy BMI. We did not observe an effect modification when fat mass and percent fat were modeled as the outcomes, and this should be further examined in larger studies. From a public health perspective, it is promising that higher childhood BMI accompanying a high maternal pre‐pregnancy BMI appears modifiable by PA, at least in boys.

Some previous studies have observed a positive association between birth weight and subsequent risk of obesity,^10^ whereas others have suggested a possible U‐shaped relationship.^29^ We did not observe any associations between birth weight for gestational age and body composition in childhood. This is similar to the results from Chomtho et al,^30^ but contrary to other studies.^14^, ^31^ Eriksson et al^12^ observed an association between birth weight and fat mass in boys, but not in girls. Neither of the studies observed an association between birth weight and measures of fat relative to fat‐free or total body mass,^12^, ^14^, ^30^, ^31^ which is possibly explained by the association between birth weight and larger size in general, rather than specifically an association with adiposity.

However, we observed a stronger relationship between birth weight and BMI in girls than in boys, which is in agreement with a previous study that showed a stronger association between birth weight and subsequent risk of obesity in girls than in boys.^29^ The lack of effect modification by PA on the association between birth weight and childhood adiposity is consistent with the results from Ridgway et al,^18^ but in contrast to Boone‐Heinonen et al 19 who observed an effect modification in girls. However, none of these studies adjusted their analyses for gestational age. Thus, these results may therefore be confounded by natural high and low birth weight from being born before or after term. However, we cannot exclude a genetic contribution to low or high birth weight, that is, birth weight for gestational age may not be an adequate measure of intrauterine growth.^32^

Infants who have experienced growth restraint in utero, often measured by a low birth weight, tend to gain weight rapidly—a so‐called catch up growth.^33^ Rapid infant weight gain is consistently associated with higher fat mass 34 and has been considered one of the most important early life risk factors for obesity.^35^ We observed a significant interaction by sex suggesting a stronger association in boys compared to girls. Furthermore, there were significant interactions with both MVPA and VPA on the associations between infant weight gain with fat mass and percentage body fat in boys (Table 2).

Figure 2 indicates that boys with a rapid infant weight gain may be more vulnerable to an inactive lifestyle, and that high‐intensity PA may mitigate, although not eliminate, the influence of rapid infant weight gain on adiposity. An increase in *z*‐score equal to or larger than 0.67 is commonly referred to as upward centile crossing and defined as rapid infant weight gain, as 0.67 represents the difference between each displayed percentile line on standard infant growth charts (ie, 2nd, 10th, 25th, 50th, 75th, 90th, and 98th percentile lines).^36^, ^37^ According to these definition criteria, 18.7% of the girls and 22.5% of the boys in the present study have had a rapid infant weight gain. Our results indicate that boys who gained weight rapidly in infancy but being in the 75th percentile of VPA in childhood reduce the predicted fat mass by more than 1 kg compared to those being in the 25th percentile of VPA. For the low active boys below the median for MVPA, an increase in weight *z*‐score of 0.67 is associated with 1.55 kg higher fat mass (*B* = 2.32 × 0.67) in childhood. Given the average fat mass in this sample of 10.0 kg, an increase in this magnitude is noteworthy. Neither MVPA nor VPA modified the association between rapid infant weight gain and BMI, which may be explained by the inability of BMI to discriminate between fat mass and fat‐free mass, where fat‐free mass represents the largest component (in average 29.5 kg compared with 10.0 kg fat mass, Table 1). This underscores the importance of including valid measures of body composition when examining determinants of childhood obesity. The results in the present study are contrary to the study by Kolle et al,^20^ in which no effect modification of MVPA was observed on the association between infant conditional weight gain (0‐2 years) and fat mass in 30‐year‐olds (both sexes combined). Thus, it is unclear whether the effect modification by MVPA and VPA observed in this study persists into adulthood.

The underlying mechanism for the association between rapid infant weight gain and subsequent development of obesity is not clear, and some have suggested that it may be that increased growth simply results in larger size.^1^ Another proposed theory is that an undernourished prenatal environment leads to developmental responses to which the fetus anticipates it may be exposed to after birth, possibly resulting in a mismatch between the prenatally undernourished and postnatally nourished environments, thus leading to a rapid infant weight gain and increased risk of adult disease and obesity.^4^, ^38^ We adjusted our analyses for birth weight. In combination with previous findings suggesting that birth weight does not modify the association between infant weight gain and childhood BMI,^6^ it appears that rapid infant weight gain, at least in boys, is associated with increased risk of an unfavorable body composition regardless of birth weight. The question arises whether the underlying mechanism for development of later adiposity is the same for those who were growth restricted in utero and thereafter rapidly gained weight during infancy, compared to those who experienced a rapid infant weight gain without previous in utero growth restriction? Thus, it would be interesting to examine if PA modifies the association between rapid infant weight gain and later adiposity similarly in these two groups.

The observed sex differences may be explained by differences in early developmental responses in boys and girls.^39^, ^40^ Our results suggest that boys may be more vulnerable to high maternal BMI and rapid weight gain than girls, and that elevated fat mass and BMI due to early life risk factors can be more easily reduced by MVPA and/or VPA in boys. Other possible explanations are differences in pubertal status between boys and girls and differences in PA levels. A high amount of PA appears necessary to attenuate the increased risk related to a high maternal pre‐pregnancy BMI and rapid infant weight gain, and on average, boys are more likely to accumulate high amounts of MVPA and VPA. Lastly, due to the small sample size, we cannot exclude possible significant associations and an effect modification by PA in girls.

### 4.1 Strengths and limitations

This study should be interpreted keeping some strengths and limitations in mind. Although we consider it a strength that we examined associations between several early life risk factors on both body composition and BMI, the number of statistical tests increases the risk of a chance findings. Therefore, we consider our study explorative in nature aiming to suggest avenues for future research. The small sample size limited the possibility for a more refined stratification into tertiles or quartiles of PA when significant interactions between the main exposure and PA were observed. Sample size also limited the possibility to include additional covariates in our models, for example diet. It is likely that highly active children may also eat more healthily. Low statistical power can lead to type II errors, and we therefore examined associations using BMI as the outcome variable with a larger sample size. To compensate for the low number of participants in some of our analyses, we conducted multiple imputations. However, this does not fully address the issue of low sample size. Another important limitation is that body composition and BMI were measured at the same time point as PA. Ideally, multiple measures of PA conducted during childhood may increase the possibility to infer causality. The study sample is active and healthy, that is, only 1.3% of the children were classified as obese and the majority satisfied the recommended PA level of ≥60 min/d of MVPA. However, activity levels are similar to that observed in representative samples of Norwegian 9‐ to 15‐year‐olds.^41^ Furthermore, although the vast majority of the participants have highly educated parents, there were no differences between participants and nonresponders concerning parental education, birth weight, maternal pre‐pregnancy BMI, maternally reported PA at age 7 and children's BMI at age 7 (data not shown). Nevertheless, we cannot exclude the possibility of selection bias, which is not unfamiliar in epidemiological studies 42 and may influence the generalizability of the results.

Our objective measures of body composition by DXA and PA by accelerometry are strengths of this study, providing an opportunity to examine the modifying effect of both moderate and vigorous intensity PA on early life and childhood obesity associations.

### 4.2 Perspective

Prevention of obesity is an important public health goal, and although high intensity PA is associated with lower adiposity in young children,^16^ it is unclear whether PA attenuates the increased risk of childhood obesity accompanying high maternal pre‐pregnancy BMI, high birth weight and rapid infant weight gain.^18^, ^19^, ^20^ The results from this study in Norwegian children suggest that the associations between maternal pre‐pregnancy BMI and rapid infant weight gain with fat mass and BMI in 9‐ to 12‐year‐olds are attenuated by higher MVPA and VPA in boys, but not in girls. We observed no association between birth weight for gestational age and childhood body composition, nor modified by level of MVPA or VPA.

High‐intensity PA may be considered as one of many public health strategies to curb childhood obesity, especially in boys who are prone to obesity due to high maternal pre‐pregnancy BMI and rapid infant weight gain. These results should be confirmed in larger studies.
