# Supplementary material for: Early life risk factors for childhood obesity—Does physical activity modify the associations? The MoBa cohort study
Source: Scand J Med Sci Sports. 2019 Jul 3;29(10):1636–46. doi: 10.1111/sms.13504 (PMC6852336; doi:10.1111/sms.13504)
Supplement: Supplementary file 1 [file SMS-29-1636-s001.pdf]

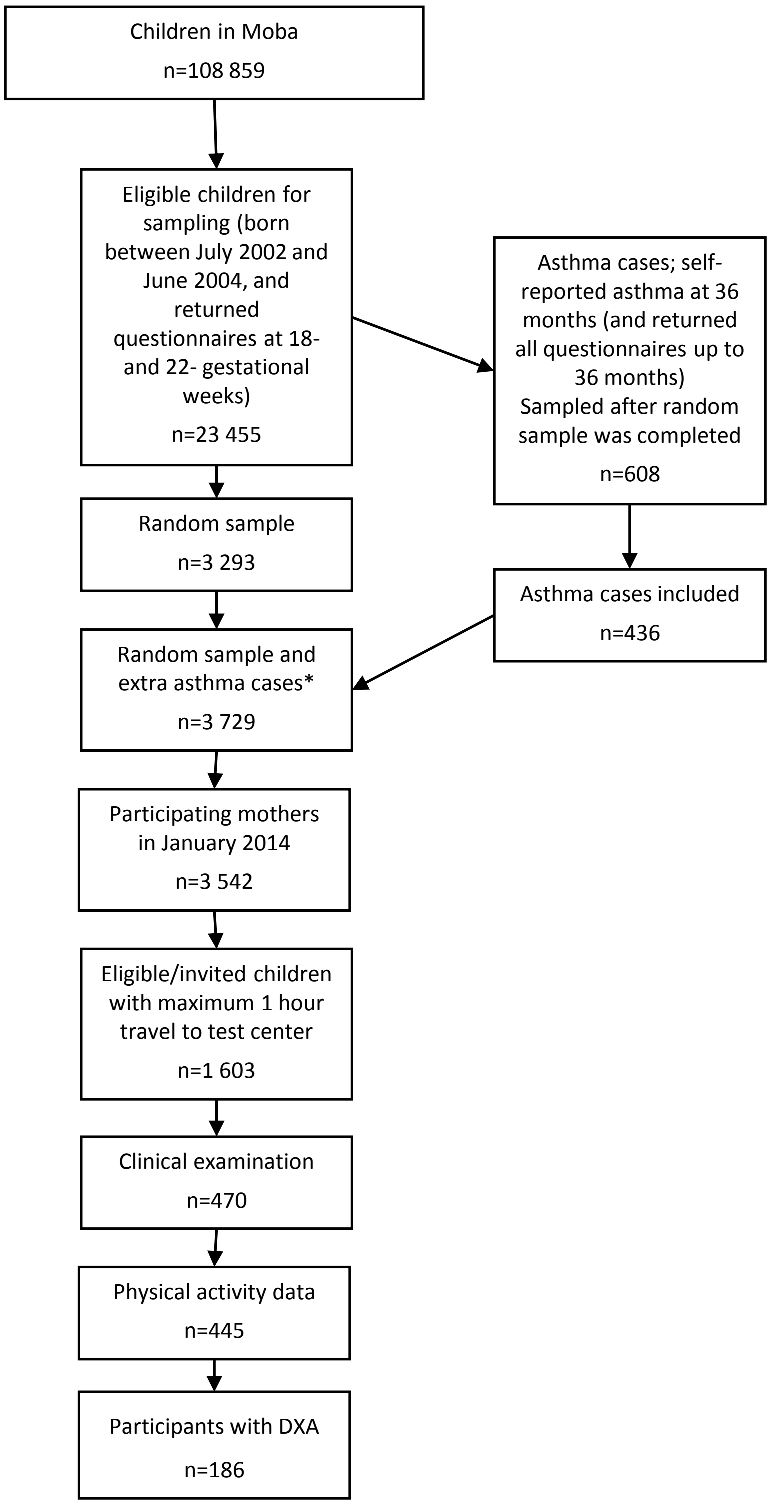

Figure S1: Flow-chart of study participants

DXA- Dual-energy X-ray absorptiometry

*Among the eligible children (n=23 455) a random sample were drawn (n=3 293) , and thereafter extra asthma cases were sampled (n=608).

Early life risk factors for childhood obesity-does physical activity modify the associations?:The Norwegian Mother and Child Cohort Study

Guro Pauck Bernhardsen[1], Trine Stensrud[1], Wenche Nystad[2], Knut Eirik Dalene[1], Elin Kolle[1] and Ulf Ekelund[12]
[1]Norwegian School of Sports Sciences, Department of Sport Medicine, Oslo Norway; [2]Norwegian Institute of Public Health, Department of Non-communicable Diseases, Oslo, Norway.
Corresponding author: Guro Pauck Bernhardsen, Work phone: +47 23262293, e-mail: g.p.bernhardsen@nih.no
