# Supplementary material for: Early life risk factors for childhood obesity—Does physical activity modify the associations? The MoBa cohort study
Source: Scand J Med Sci Sports. 2019 Jul 3;29(10):1636–46. doi: 10.1111/sms.13504 (PMC6852336; doi:10.1111/sms.13504)
Supplement: Supplementary file 2 [file SMS-29-1636-s002.pdf]

**Supporting Information File 1 (FileS1): Information on imputation method, number of missing values, participants with complete and incomplete data and complete case analyses.**

We assume that data are missing at random (MAR), given the observed variables that are included in the imputation model.

We used Fully Conditional Specification (FCS), with predictive mean matching (with 5 of the closest observations to draw from) when imputing on missing values on maternal pre-pregnancy weight, birth weight, infant weight gain and interactions terms and ordered logistic regression for parental education. We performed separate imputation models for boys and girls. Number of missing values in each exposure/covariate with missing, and descriptive statistics in the complete and imputed variable are presented in S2 File- Table 2.

All variables in the full models were included in the imputation model, in addition to the auxiliary variables= child's weight at 6 weeks, child's weight at 6 months, child's weight at 15 months, maternal weight by the end of pregnancy, maternal weight 6 months postpartum, test center and childhood waist circumference. Interaction terms (early life risk factor x MVPA/VPA) were included in the imputation model as "just another variable" (JAV). We removed participants with missing on the outcome of interest for each analysis model.

FileS1- Table S1: Descriptive characteristics (mean and sd) of study participants, stratified by sex and participants with complete and incomplete data.

| | BOYS | | GIRLS | |
|---|---|---|---|---|
| | Complete data (n=192) | Incomplete data (n=50) | Complete data (n=166) | Incomplete data (n=37) |
| | Mean (sd) | Mean (sd) | Mean (sd) | Mean (sd) |
| MVPA (min/day) | 73.0(26.5) | 77.6(26.6) | 58.5(18.5) | 56.6(21.0) |
| VPA (min/day) | 29.4(15.2) | 29.9(12.2) | 21.3(10.1) | 20.8(11.2) |
| Fat mass (kg) | 10.1(4.1) | 9.5(4.4) | 11.0(3.2) | 13.9(7.9)* |
| Fat free mass (kg) | 29.3(3.5) | 29.6(3.9) | 28.3(4.5) | 29.8(5.9) |
| Percent fat (%) | 24.9(6.3) | 23.5(5.7) | 27.6(4.7) | 30.2(8.8) |
| BMI (kg/m$^2$) | 17.8(2.4) | 18.1(2.6) | 17.6(2.0) | 18.5(3.2) |
| Maternal pre-pregnancy BMI (kg/m$^2$) | 23.5(3.9) | 25.6(5.2)* | 23.6(4.0) | 24.8(4.0) |
| Birth weight (z-score) | 0.11(1.03) | 0.23 (1.09) | 0.01(1.05) | 0.27(1.16) |
| Infant weight gain (z-score) | -0.07(1.11) | -0.74(0.90) | -0.07(1.04) | -0.31(0.58) |

*p<0.05 for differences between participants with complete- and incomplete data

FileS1- Table S2: Covariates and exposures with missing values and descriptive statistics of complete variables (complete) and the variables with imputed on missing values (MI).

| Variable | n missing (%) | Complete | MI |
|---|---|---|---|
| Boys | | | |
| Parental education (%) | 3(1%) | | |
| *<High school* | | 2.5% | 2.5% |
| *High school* | | 20.9% | 20.8% |
| *College/university 1-4years* | | 39.7% | 39.7% |
| *College/university >4years* | | 36.8% | 36.9% |
| Maternal pre-pregnancy BMI (kg/m$^2$) mean(range) | 6 (2%) | Mean: 23.9<br>Range: 17.0-40.23 | Mean: 23.9<br>Range: 17.0-40.23 |

| | | | |
|---|---|---|---|
| Birth weight (kg) | 1 (<1%) | Mean: 3.7<br>Range: 1.10-5.45 | mean: 3.7<br>range: 1.10-5.45 |
| Birth weight[a] (z-score) | 1 (<1%) | Mean: 0.14<br>Range: -3.60 - 3.63 | mean: 0.14<br>range: -3.60 - 3.63 |
| Infant weight gain (z-score) | 42(17%) | Mean: -0.093<br>Range: -3.64 - 3.92 | Mean: -0.105<br>Range: -3.64 - 3.92 |
| Maternal BMI x MVPA | 6(2%) | Mean: 1764.2<br>Range: 295.9-4871.5 | Mean: 1759.7<br>Range: 295.9-4871.5 |
| Maternal BMI x VPA | 6(2%) | Mean: 698.7<br>Range: 83.6-2106.4 | Mean: 697.5<br>Range: 83.6-2106.4 |
| Birth weight[a] x MVPA | 1(<1%) | Mean: 7.2<br>Range: -377.0 – 270.6 | Mean: 7.2<br>Range: -377.0 – 270.6 |
| Birth weight[a] x VPA | 1(<1%) | Mean: 2.18<br>Range: -233.9 – 132.5 | Mean: 2.21<br>Range: -233.9 – 132.5 |
| Weight gain x MVPA | 42(17%) | Mean: -6.86<br>Range: -452.0 – 384.4 | Mean: -6.82<br>Range: -452.0 – 384.4 |
| Weight gain x VPA | 42(17%) | Mean: -2.91<br>Range: -204.4 – 130.6 | Mean: -2.96<br>Range: -204.4 – 130.6 |
| Girls | | | |
| Parental education<br>*<High school*<br>*High school*<br>*College/university 1-4years*<br>*College/university >4years* | 3 (1%) | <br>2.0%<br>24.0%<br>40.0%<br>34.0% | <br>2.0%<br>23.8%<br>40.0%<br>34.2% |
| Maternal pre-pregnancy BMI (kg/m$^2$) | 6(3%) | Mean: 23.8<br>Range: 14.7-39.6 | Mean: 23.8<br>Range: 14.7-39.6 |
| Birth weight (kg) | 0(0%) | - | - |
| Birth weight[a] (z-score) | 0(0%) | - | - |
| Infant weight gain (z-score) | 32(16%) | Mean: -0.081<br>Range: -3.25 – 3.94 | Mean: -0.084<br>Range: -3.25 – 3.94 |
| Maternal BMI x MVPA | 6(3%) | Mean: 1376.3<br>Range: 382.1-3153.5 | Mean: 1372.6<br>Range: 382.1-3153.5 |
| Maternal BMI x VPA | 6(3%) | Mean: 501.2<br>Range: 80.9-1700.2 | Mean: 499.3<br>Range: 80.9-1700.2 |
| Birth weight[a] x MVPA | 0(0%) | - | - |
| Birth weight[a] x VPA | 0(0%) | - | - |
| Weight gain x MVPA | 32(16%) | Mean: -5.72<br>Range: -172.7- 313.5 | Mean: -5.64<br>Range: -172.7- 313.5 |
| Weight gain x VPA | 32 (16%) | Mean -2.02<br>Range: -66.02- 105.7 | Mean: -2.03<br>Range: -66.02 – 105.7 |

[a]Birth weight for gestational age (z-score)

FileS1 - Table S3: Association (unstandardized regression coefficients with 95%CI) between physical activity (MVPA/VPA) and early life risk factors with body composition and BMI in childhood, and interaction between early life risk factors and MVPA/VPA. Complete case analyses.

| | Fat mass (kg) | | Fat free mass (kg) | | Percent fat (%) | | BMI (kg/m²) | |
|---|---|---|---|---|---|---|---|---|
| | Boys | Girls | Boys | Girls | Boys | Girls | Boys | Girls |
| MVPA (min/day) | -0.03(-0.06,0.00) | -0.03(-0.09,0.02) | -0.01(-0.03,0.02) | -0.01(-0.07,0.04) | -0.04(-0.09,0.00) | -0.03(-010,0.4) | -0.01(-0.03,-0.00) | -0.00(-0.02,0.01) |
| VPA (min/day) | -0.05(-0.11,0.00) | -0.05(-0.16,0.06) | -0.00(-0.05,0.04) | -0.01(-0.12,0.10) | -0.09 (-0.17, -0.01) | -0.05(-0.19.0.09) | -0.04(-0.06,-0.01) | -0.01(-0.04,0.02) |
| Maternal pre-pregnancy BMI (kg/m²) | 0.44(0.25,0.62) | 0.19(-0.06,0.43) | 0.14(0.03,0.25) | 0.06(-0.08, 0.20) | 0.62(0.32,0.91) | 0.25(-0.11,0.60) | 0.28(0.22,0.35) | 0.09(0.01,0.17) |
| Maternal pre-pregnancy BMI xMVPA | -0.001(-0.02, 0.00) | -0.001, -0.02,0.01) | -0.005(-0.01,0.00) | -0.003(-0.01, 0.01) | -0.010(-0.03,0.00) | 0.005(-0.02,0.03) | -0.001(-0.00, 0.00) | -0.000(-0.00, 0.00) |
| Maternal pre-pregnancy BMI xVPA | -0.012(-0.03, 0.00) | -0.001(-0.03,0.03) | -0.004(-0.01 ,0.00) | -0.003(-0.02, 0.01) | -0.018(-0.04, 0.01) | 0.007(-0.03, 0.05) | -0.005(-0.01, 0.00) | -0.002(-0.01,0.01) |
| Birth weight for gestational age (z-score) | 0.19(-0.53,0.92) | 0.23(-0.60,1.06) | 0.17(-0.26,0.60) | 0.37(-0.10,0.83) | 0.22(-0.92,1.37) | 0.16(-1.03,1.35) | 0.16 (-0.12,0.43) | 0.32(0.02,0.63) |
| Birth weight x MVPA | 0.009(-0.01, 0.03) | 0.020(-0.03, 0.07) | -0.000(-0.01, 0.01) | 0.019(-0.01, 0.05) | 0.028(-0.01, 0.06) | 0.041(-0.04, 0.12) | 0.000(-0.01, 0.01) | 0.001(-0.01, 0.02) |
| Birth weight x VPA | 0.007(-0.03,0.04) | -0.002(-0.12, 0.11) | 0.003(-0.02, 0.02) | 0.043(-0.02, 0.11) | 0.031(-0.02, 0.08) | 0.021(-0.14,0.19) | 0.001(-0.01, 0.01) | -0.004(-0.04, 0.03) |
| Infant weight gain (z-score) | 1.29(0.36,2.21) | 0.08(-0.80, 0.96) | 0.82(0.30,1.35) | -0.04(-0.62,0.53) | 1.87(0.39,3.34) | 0.09(-1.37,1.54) | 0.75(0.37,1.13) | 0.32(-0.04,0.68) |
| Infant weight gain x MVPA | -0.025(-0.04,-0.00) | -0.005(-0.04,0.03) | -0.002(-0.01, 0.01) | 0.000(-0.02, 0.02) | -0.031(-0.06,0.00) | -0.013(-0.07,0.05) | -0.002(-0.01, 0.01) | 0.002(-0.01,0.02) |
| Infant weight gain x VPA | -0.059(-0.10,-0.02) | -0.007(-0.09, 0.08) | -0.004(-0.03, 0.02) | -0.005(-0.06, 0.05) | -0.084(-0.14,0.02) | -0.025(-0.16, 0.11) | -0.007(-0.02, 0.01) | 0.007(-0.03, 0.04) |

BMI- Body Mass Index; MVPA- Moderate to vigorous physical activity; VPA- Vigorous physical activity,

**Number of participants in complete case analyses (boys/girls):**

Body composition (fat mass, fat free mass, percent fat) - MVPA: 97/86

Body composition (fat mass, fat free mass, percent fat) - VPA: 97/86

Body composition (fat mass, fat free mass, percent fat) - maternal pre-pregnancy BMI: 94/82

Body composition (fat mass, fat free mass, percent fat) - birth weight: 94/82

Body composition (fat mass, fat free mass, percent fat) - infant weight gain: 82/73

BMI- MVPA/VPA: 239/200

BMI- maternal pre-pregnancy weight: 232/195

BMI- birth weight: 232/195

BMI- infant weight gain: 197/169
